# One patient - three head and neck primaries: nasopharyngeal, tongue and thyroid cancers

**DOI:** 10.1186/1756-0500-6-432

**Published:** 2013-10-28

**Authors:** Muhammad Mohsin Fareed, Abdullah Al Amro, Yasser Bayoumi, Khalid HA AlQahtani, Hanadi A Fatani, Mutahar Ali Tunio, Farhan Khalid

**Affiliations:** 1 Department of Radiation Oncology, King Fahad Medical City, Riyadh 11525, Saudi Arabia; 2 Department of Otolaryngology-Head & Neck Surgery, College of Medicine, King Saud University, Riyadh, Saudi Arabia; 3 Department of Histopathology King Fahad Medical City, Riyadh 11525, Saudi Arabia

**Keywords:** Nasopharyngeal carcinoma, Tongue cancer, Papillary thyroid carcinoma, Metachronous cancers, Head and neck cancers, Concurrent chemoradiation

## Abstract

**Background:**

We report a rare case of three head and neck malignancies in one patient. Squamous cell carcinoma of tongue and papillary thyroid carcinoma occurred as metachronous cancers in a patient with primary nasopharyngeal carcinoma. These three pathologically distinct malignancies of head and neck region in one patient is a rare phenomenon and is not reported so far.

**Case presentation:**

A 60 year old Saudi female patient presented in March 2011 with locally advanced nasopharyngeal carcinoma. After completion of concurrent chemoradiation in June 2011, she developed two new primaries i-e thyroid cancer and tongue cancer in May 2012 along with recurrent nasopharyngeal carcinoma. We discuss histopathologic features, diagnostic tools and treatment modalities for this rarely existing case.

**Conclusion:**

High index of suspicion and thorough work up is essential in follow up of patients with head and neck primary cancers. The effect of field cancerization and environmental factors need to be explored in greater depths in such selected cases. However, which patients are at increased risk of triplet primaries, is still unknown.

## Background

The incidence of three primary cancers in one patient, whether synchronous (simultaneous) or metachronous (successive) is rare, especially if it comes to malignancies appearing in head and neck region. It is always important for the treating oncologists to keep in mind the possibility of a synchronous or a metachronous malignancy in a cancer patient. Patients with head and neck primary cancers have increased propensity of having second cancers especially in patients with tongue, pyriform sinus, larynx, oral cavity and tonsillar cancers. Schwartz et al., observed 19% incidence of second cancers in the same region in patients presenting with primary head and neck cancers. Among them, 41% were synchronous and 59% were metachronous [1]. As the development of a second malignancy is almost always fatal, there is a need for additional efforts in work up, radiation treatment planning, goals of treatment and follow up schedules in such a group of patients [1,2].

Although there have been reports of second cancers arising within the context of primary head and neck cancer, the incidence of three head and neck primary cancers is extremely rare, if not extinct. Among head and neck tumors, nasopharynx, larynx and hypopharynx have been described as having increased incidence of triplicate malignancies [3].

Our case is therefore worth mentioning as it reports three different primary cancers in a single patient arising in nasopharynx, tongue and thyroid. Interesting was the fact that these three primaries were with three different histologies: Undifferentiated carcinoma of nasopharynx, squamous cell carcinoma of tongue and papillary carcinoma of thyroid. The incidence of multiple primary malignant neoplasms increases with age and they are encountered more frequently nowadays than before owing to better diagnostic approaches. Although the scattered reports of developing three primaries have been described for other body sites, this is by far the first case mentioning three different malignancies in head and neck region [4].

Here-in we report a 60 year old lady having three primary head and neck malignancies, i.e. nasopharyngeal, tongue and papillary thyroid carcinoma.

## Case presentation

### Case report

A 60 years Saudi lady, known diabetic and hypertensive, presented on March 10, 2011 to Otorhinolaryngology department of King Fahad Medical City Riyadh. She had history of left sided nasal obstruction, decreased left sided hearing and blood tinged sputum for last 3 months. Nasendoscopy showed left sided nasopharyngeal mass, biopsy of which showed undifferentiated carcinoma on March 20^th^, 2011 Figure 1. She underwent complete staging work up including computed tomography (CT) of head & neck, chest and abdomen which showed predominantly left nasopharyngeal mass infiltrating into the parapharyngeal space without skull base or intracranial extension along with left internal jugular and spinal accessory lymph nodes Figure 2. The thyroid gland was slightly enlarged with a few bilateral calcified nodules likely representing a multinodular goiter. Further neck ultrasonography showed similar thyroid gland findings due to almost negligible index of suspicion for malignancy. Thyroid function tests (TFTs), serum thyroglobulin (TG) and serum calcium levels were within normal limits. FNAC of thyroid nodules was not performed at that time due to almost negligible index of suspicion for malignancy. Remaining staging work up was negative for distant metastases. After being discussed in multidisciplinary tumor board meeting, she was staged as T2N1M0 nasopharyngeal carcinoma and referred to Radiation Oncology and Medical Oncology departments for concurrent chemoradiation (CCRT) after dental clearance and audiology assessment. She completed 70 Gy/35 fractions using intensity modulated radiation therapy along with weekly Cisplatin 40 mg/m^2^ on June 18^th^, 2011 Figure 3. During CCRT course of treatment was tolerated with GI skin desquamation and mucositis. After the completion of treatment, at 3 months there was complete resolution of primary nasopharyngeal mass and neck nodes as evidenced by endoscopy, CT and magnetic resonance imaging (MRI).

**Figure 1:**
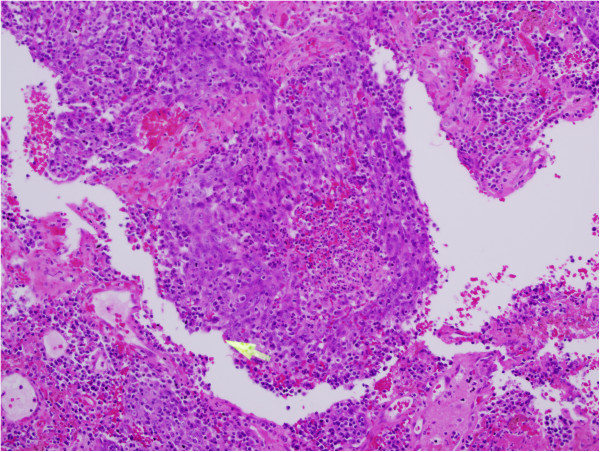
**Undifferentiated nasopharyngeal carcinoma WHO G-III.**

**Figure 2:**
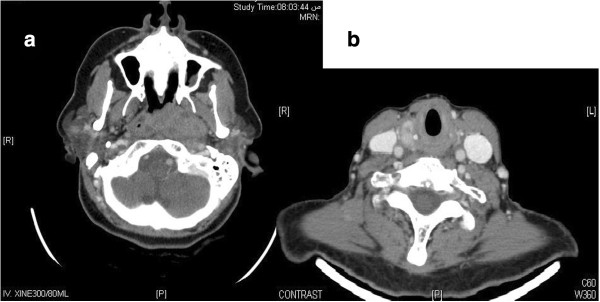
CT showing (a) Left sided nasopharyngeal tumor infiltrating into the left parapharyngeal space and abutting the left internal carotid artery and (b) Thyroid nodule right lobe.

**Figure 3:**
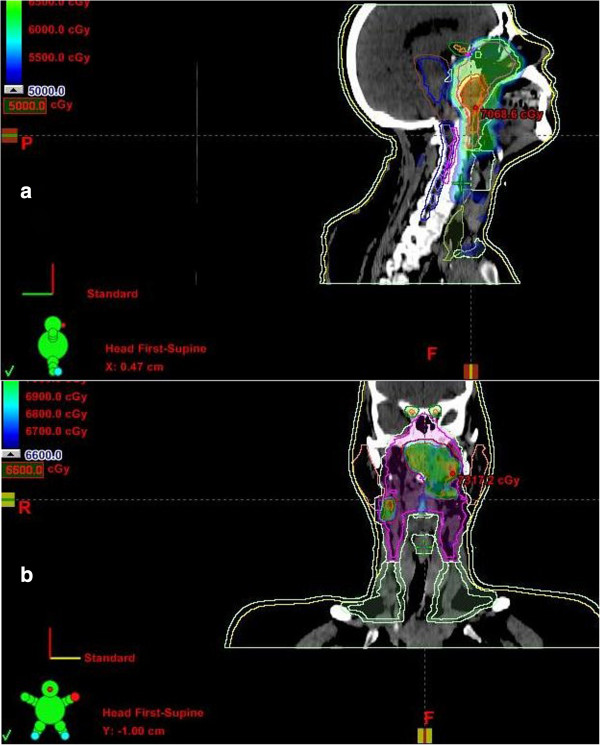
**Intensity modulated radiation therapy (IMRT) for nasopharyngeal carcinoma showing isodose distribution (a) 50 Gy and (b) 70 Gy.**

**Figure 4:**
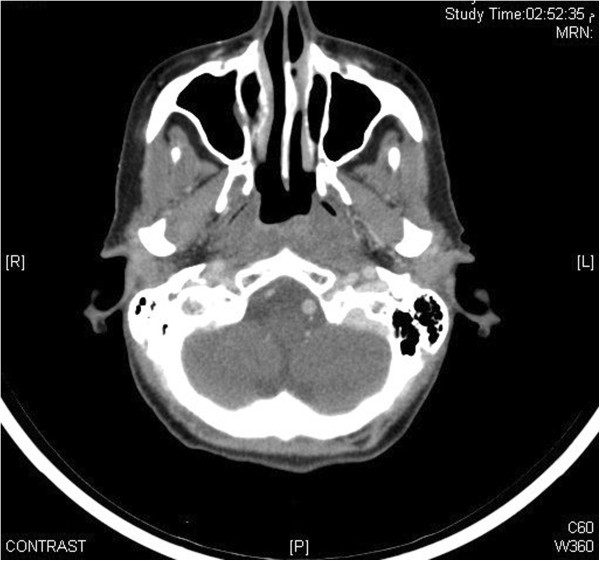
CT head and neck showing recurrent left nasopharyngeal wall mass with some localized contrast enhancement and bulging of mucosal contour without any obvious cervical lymphadenopathy.

**Figure 5:**
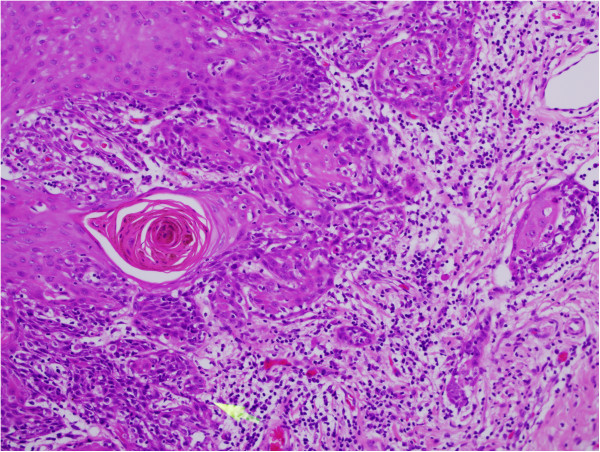
**Moderately differentiated**, **multifocal squamous cell carcinoma tongue.**

On May 6^th^, 2012, during her routine follow up, she complained of ulcer over left tongue increasing in size over 2 months and was painful even after taking analgesics. On examination, there was 1 cm ulcer over left lateral tongue without any palpable lymphadenopathy (no clinical photograph available as she refused) and delayed GII radiation induced xerostomia. CT head and neck showed new fullness along left nasopharyngeal wall with some localized contrast enhancement and bulging of mucosal contour without any obvious cervical lymphadenopathy and multiple heterogeneous nodules with dystrophic calcification seen within both thyroid lobes Figure 4. Impression was that of a possibility of a recurring local tumor. For left sided tongue lesion, excisional biopsy was performed due to its small size on June 9^th^, 2012 which showed invasive squamous cell carcinoma Figure 5. Clinical stage for tongue squamous carcinoma was labeled as cT1N0M0. FNAC from nasopharynx confirmed recurrent nasopharyngeal carcinoma of undifferentiated type. On June 12, 2012, ultrasound guided FNAC of thyroid nodule was also done which revealed papillary thyroid carcinoma. Magnetic resonance imaging (MRI) of head and neck was performed which showed previously noted nasopharyngeal fullness increasing in size along with well-defined mass tissue lesion at the posterior and superior wall of the nasopharynx measuring 22 ×20 mm bulging into the nasopharyngeal airway. Also infiltrating enhancing lesion along the left border of the tongue was noted and enlarged thyroid gland with multiple nodules. There was no cervical lymphadenopathy or other distant metastases.

**Figure 6:**
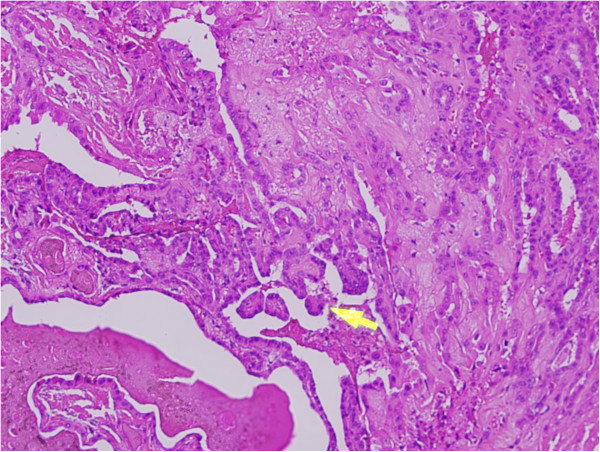
**Papillary thyroid carcinoma showing growth pattern represented by finger**-**like projections lined by neoplastic cells.**

**Figure 7:**
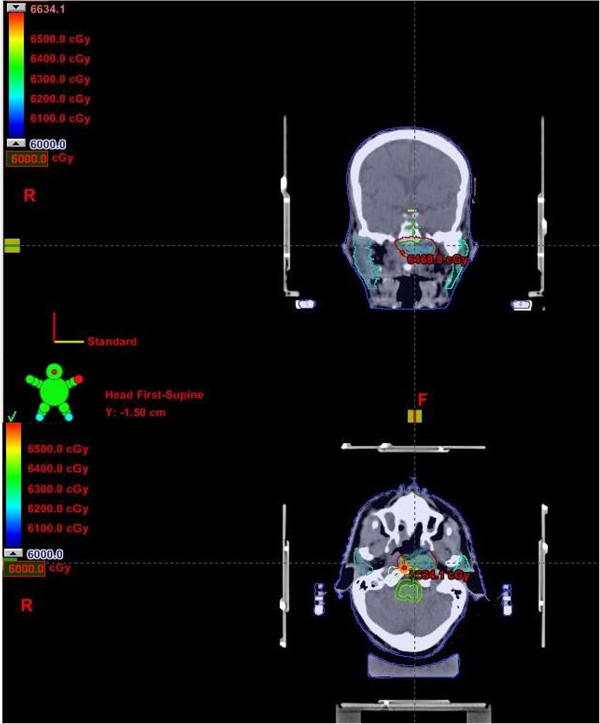
Stereotactic radiation therapy for recurrent nasopharyngeal carcinoma.

On June 17^th^ 2012, her case was discussed again in multidisciplinary tumor board meeting and the panel of experts agreed to go for surgery of three sites. On August 11^th^ 2012, she underwent total thyroidectomy, left partial glossectomy with left lateral and central neck dissection and nasopharyngeal tumor resection. In partial glossectomy, margins were sent as fresh frozen. All the margins came out negative; except the anterior margin which showed mild to moderate dysplasia. Detailed histopathology report showed multifocal moderately differentiated squamous cell carcinoma of the tongue, 1.1 cm in maximum dimension, depth of invasion 2 mm, negative perineural and lymphovascular invasion, 0/12 lymph nodes (pT1N0). Nasopharyngeal mass was positive for undifferentiated nasopharyngeal carcinoma, WHO Grade –III, while thyroid specimen showed papillary thyroid carcinoma, conventional type, tumor size 2.2 cm in maximum dimension, positive extrathyroid extension in soft tissue, absent lymphovascular invasion, and 0/7 lymph nodes Figure 6; tumor was about 1 mm from inked resection margin (Thyroid carcinoma staged as T2N0M0). On November 13^th^ 2012, she was given 100 milli curie (mCi) radioactive iodine. Seven-day postablation whole body scintigraphy revealed two foci of post-thyroidectomy residual functioning thyroid tissue in the neck. No distant metastatic lesions were noted. Recurrent nasopharyngeal primary was treated with stereotactic radiotherapy with total dose 60 Gy in 30 fractions that completed on January 01, 2013. Figure 7. At six months after re-irradiation she was found asymptomatic with GII late xerostomia.

## Discussion

Multiple primary cancers are a rare entity whose etiology is not well understood although some pathogenic factors like persistent carcinogenic influence, field cancerization, increasing use of systemic chemotherapy or radiotherapy, hormonal manipulation, targeted therapy, genetic alteration, immune suppression, tissue transplantation and improved survival are implicated. The incidence of second primary synchronous or metachronous tumor is increasing and reported as high as 10%. A meta analyses shows the frequency of second primary tumor as 3-5%, a third primary tumor as 0.5%, and a fourth primary tumor as 0.3% [5].

The second tumor is called synchronous if it is found simultaneously or within six months of the diagnosis of primary. All malignancies found later than six months after the primary are considered as metachronous [6]. In a study by Panosetti et al. on 855 patients of multiple primary head and neck cancer patients, 42% had synchronous while 58% had metachronous tumors. Survival rates varied according to the treatment as evident by the fact that 5 year survival was only 8% among those synchronous tumors in whom treatment was modified whereas it was 28% in patients with unmodified treatment plans [7]. Another study done on 796 patients showed that second primaries were mostly found in esophagus, hypopharynx, buccal cavity, and the lung. Incidence of multiple cancers and synchronous primaries were more prevalent in prospective studies rather than in retrospective series. The five-year survival rate among patients with synchronous cancers in these two studies was 18% as compared to those with metachronous cancers (41-55%). The prognosis for synchronous cancers deteriorated if the planned treatment had to be modified following the discovery of a second primary.

Individuals with a history of multiple cancers should have a complete family history evaluation and follow-up for development of subsequent primaries. Genetic counseling, risk estimation, and cancer screening must be emphasized. Every subsequent occurring tumor must be biopsied [1]. There are different diagnostic modalities to consider in such cases. Systematic pretherapeutic panendoscopy should be used to help detect early asymptomatic second primaries which might be missed during clinical examination [8]. Because of the relatively small proportion of malignant findings and lack of convincing data on its effect on survival rates, the value of panendoscopy has been debated. However, its significance is often crucial for the individual patient, despite the low proportion of positive findings. The study by Hujala K. et al. included 203 consecutive patients with squamous cell cancer of the upper aerodigestive tract who underwent panendoscopy in Turku University Central Hospital as part of the initial diagnostic workup from 1992–1999. Eight patients with synchronous second primaries and 19 patients with metachronous tumors were diagnosed [9]. Haughty et al. recommended routine interval endoscopic intervention within 2 years of treatment for optimum detection of second primaries in head and neck cancer patients. Also, a lifetime of clinical surveillance is suggested for aerodigestive tract second neoplasms in oral cavity, oropharynx, and hypopharynx cancer patients and for lung and non-aerodigestive tract neoplasms in larynx cancer patients based on their meta-analysis of second malignant tumors in head and neck cancers. Head and neck second primary tumors were more common in oral cavity, oropharynx and hypopharynx [10].

In the long run, prevention has to play a decisive role in the fight against second primary tumors of the upper aerodigestive tract. Possible improvements in early diagnosis, genetic examinations, information campaigns as well as research of carcinogenic environmental pollutants are of foremost interest to the clinician [11]. Screening programs and chemoprevention strategies should be directed toward cancer patients with initial head and neck malignancies [9]. The effect of first tumor on the 2nd primary or vice versa is still not fully understood and need to be explored. The second primary tumor is usually more aggressive, more treatment resistant, and metastasizes early, requiring a more aggressive treatment strategy [5].

Our patient had metachronous tumors (tongue cancer and thyroid cancer) arising almost one year after the primary diagnosis of nasopharyngeal carcinoma. It was associated with recurrence at the primary site also. It emphasizes the need for vigilant, regular and meticulous follow up of such patients along with appropriate imaging and biopsy techniques. The efficacy of 18F-FDG PET/CT has been discussed in detection of distant metastases in high risk head and neck primary cancers, but its role in detecting synchronous and metachronous lesions of the same region is not clear [12]. The role of panendoscopy and subsequent biopsy of suspicious lesion is more compromising. Such early suspicious lesions should be biopsied as early as possible in order to offer best curative modality to these patients. The prognosis in such patients is obviously not as good as in patients with primary cancers. The burden of interventional investigations and complication rates are also obviously high in these patients. The prognosis of synchronous tumors is significantly lower when compared to malignancies of a metachronous nature, despite some encouraging individual results. Only the early implementation of aggressive treatment methods for second primaries is successful in terms of survival [13].

## Conclusions

Three primaries in head and neck region are rare and this is by far the first case reporting metachronous thyroid and tongue cancers in a patient with primary nasopharyngeal carcinoma. Careful follow up with special emphasis on early detection of any subsequent primary tumors is very critical in patients presenting with head and neck cancers. Role of panendoscopy and PET is controversial but can be of substantial help in this regard.

## Consent

Written informed consent was obtained from the patient for publication of this case report and any accompanying images. A copy of the written consent is available for review by the Editor-in-Chief of this journal.

## Abbreviations

CT: Computed tomography; Gy: Grays; FNA: Fine needle aspiration; MRI: Magnetic resonance imaging; WHO: World health organization; mCi: Milli Curie; 18F-FDG PET: Fluorodeoxyglucose (^18^F) Positron emission tomography.

## Competing interests

The authors declare that they have no competing interests.

## Author’s contributions

MMF perceived the idea of this case report and written the manuscript. AAA is the treating radiation oncologist for nasopharyngeal carcinoma. MAA treated the thyroid cancer with radioactive iodine ablation. KQ performed thyroidectomy and neck dissection along with excision biopsy of tongue lesion. HAF performed the histological examination of nasopharynx, tongue and thyroid specimens. MAT, YB and FK helped in collecting the relevant clinical, radiologic and pathologic data. All authors read and approved the final manuscript.
